# Intracellular chemiluminescence activates targeted photodynamic destruction of leukaemic cells

**DOI:** 10.1038/sj.bjc.6603241

**Published:** 2006-07-04

**Authors:** R Laptev, M Nisnevitch, G Siboni, Z Malik, M A Firer

**Affiliations:** 1 Department of Chemical Engineering and Biotechnology, College of Judea and Samaria, Ariel 44837, Israel; 2 Faculty of Life Sciences, Bar-Ilan University, Ramat-Gan, Israel

**Keywords:** targeted drug delivery, photodynamic therapy, transferrin, luminol, intracellular activation

## Abstract

Photodynamic therapy (PDT) involves a two-stage process. A light-absorbing photosensitiser (Ps) is endocytosed and then stimulated by light, inducing transfer of energy to a cytoplasmic acceptor molecule and the generation of reactive oxygen species that initiate damage to cellular membrane components and cytolysis. The expanded use of PDT in the clinic is hindered by the lack of Ps target-cell specificity and the limited tissue penetration by external light radiation. This study demonstrates that bioconjugates composed of transferrin and haematoporphyrin (Tf–Hp), significantly improve the specificity and efficiency of PDT for erythroleukemic cells by a factor of almost seven-fold. Fluorescence microscopy showed that the conjugates accumulate in intracellular vesicles whereas free Hp was mostly membrane bound. Experiments with cells deliberately exposed to Tf–Hp at <LD_100_ doses showed that surviving cells did not develop resistance to subsequent treatments with the conjugate. Furthermore, we show that the compound luminol induces intracellular chemiluminescence. This strategy was then used to obviate the use of external radiation for Ps activation by incubating the cells with luminol either before or together with Tf–Hp. This novel chemical means of PDT activation induced cytotoxicity in 95% of cells. These combined approaches provide an opportunity to develop broader and more effective applications of PDT.

The nonselectivity of chemotherapeutic agents has stimulated development of targeted drug delivery strategies such as ligand toxin conjugates (LTCs), involving the coupling of an effector molecule to a transport ligand of a target cell-specific receptor (Rihova, 1998; Mrsny, 2004). Smaller ligands such as peptides or other small molecules may have advantages over larger molecules like antibodies (Rihova, 1998; Shadidi and Sioud, 2003). We recently reported on the use of small molecule-containing LTCs for the targeted destruction of plasma cells (Firer *et al*, 2003) suggesting that this approach may be applicable for multiple myeloma therapy. The LTC strategy for targeting tumour cells represents one focus of the present study.

The second focus involves photodynamic therapy (PDT), a two-stage procedure based on two nontoxic components that combine to induce membrane alterations leading to cytolysis. The first component is a photosensitiser (Ps) molecule, usually a porphyrin derivative, that when light-activated transfers energy to molecular oxygen, producing reactive oxygen species that cause direct damage to cellular components, particularly membrane phospholipids. Photodynamic therapy is believed to mediate tumour cell destruction by at least two additional mechanisms: destruction of tumour vascular cells and the induction of both inflammatory and immune antitumour reactions (Dougherty *et al*, 1998; van Duijnhoven *et al*, 2003). The history, mechanism of action and biomedical applications of PDT have been the subject of several comprehensive reviews (Ceburkov and Gollnick, 2000; Dolmans *et al*, 2003; Brown *et al*, 2004; Sharman *et al*, 2004).

Two major issues limit the wider application of PDT as a treatment modality. First, whereas Pss tend to accumulate in tumour tissue, their clinical use is often associated with side effects such as cutaneous photosensitivity (Vrouenraets *et al*, 2003; Sharman *et al*, 2004). To overcome this problem investigators have covalently linked the Ps to carrier molecules so as to localise the PDT effect (Klyashchitsky *et al*, 1994; Vrouenraets *et al*, 2003). One attractive carrier protein–receptor system for this purpose utilises the high-affinity interaction between the iron-transporter transferrin (Tf) and its cell-surface receptor (TfR, CD71). As all dividing cells require a continuous supply of iron for metabolism, it is not surprising that TfR is overexpressed on a variety of malignant cells (Ponka *et al*, 1998), so the Tf–TfR system has been used in several formats to target Ps compounds to different types of malignant cells (Hamblin and Newman, 1994; Rittenhouse-Diakun *et al*, 1995; Cavanaugh, 2002; Gijsens *et al*, 2002; Li and Qian, 2002). The second major issue regarding PDT is the limited tissue penetration of external laser light. Despite advances in the development of external light devices for phototherapy (Brancaleon and Moseley, 2002) and the successful clinical use of PDT for peripheral cancers (Stockfleth and Sterry, 2002; Dolmans *et al*, 2003; Moan and Peng, 2003; Brown *et al*, 2004) and in dermatology (Ceburkov and Gollnick, 2000), the treatment of internal body tissues remains limited to invasive procedures, such as the use of endoscopes. Although these procedures may involve acceptable discomfort to the patient compared to other treatments such as surgery, there are only isolated reports of attempts to develop molecular approaches to Ps activation. These studies used chemiluminescence (CL), in which the *in situ* conversion of molecular oxygen to superoxide ions and the subsequent release of light energy achieved is without electrical input or thermal output. Carpenter *et al* (1994) employed intracellular bioluminescent activation of hypericin and the subsequent destruction of equine dermal cells, whereas Phillip and Maximuke (1989) used a haematoporphyrin derivative (Photofrin II) and a multicomponent solution to induce intracellular CL in mammary adenocarcinomas. In searching for a simple means to induce intracellular CL, we turned to luminol, which has been successfully used in a variety of CL-based assays systems (Kricka, 2002). The mechanism of the CL reaction of luminol has been known for some time (White *et al*, 1964) and whereas some physico-chemical aspects of luminol activation in macrophages have been examined (Nemeth *et al*, 1999), the luminol–PDT connection has never been exploited to induce PDT cytotoxicity in tumour cells.

The present study combines these new approaches to overcome the above-mentioned limitations to PDT applications. We first demonstrate the efficacy of the targeted LTC strategy using the transferrin and haematoporphyrin (Tf–Hp) system. We then establish *in vitro* proof of principle that luminol can be used as a powerful molecular inducer of intracellular CL for the destruction of leukemic cells, overcoming the need of external light sources in PDT.

## MATERIALS AND METHODS

Hp, rabbit anti-human transferrin, goat anti-bovine serum albumin (BSA), transferrin, *N*-hydroxysuccinimide and luminol (5-amino-2-3-dihydro-1, 4-phtalazinedione) were purchased from Sigma-Aldrich Chemical Co., Rehovot, Israel, *N*,*N*-dicyclohexyl carbodiimide (DCC) and tetrahydrofuran were from Carlo Erba, Radona, Italy. Horse serum (HS), fetal calf serum, Dulbecco's modified Eagle's medium (DMEM), RPMI, L-glutamine and combined antibiotics were purchased from Biological Industries Ltd (Bet Haemek, Israel). High-pressure liquid chromatography (HPLC) solvents were from Merck, Rosh Haayin, Israel.

### Cells

U7.6 is a murine hybridoma that secretes IgG1 antibody against dinitrophenol and was a kind gift of Professor Eshhar (Weizmann Institute of Science, Rehovot, Israel). These cells as well as Friend's leukaemia (FL) cells were grown in DMEM containing 15% HS, 2 mM L-glutamine and combined antibiotics. Human K-562 cells were grown in RPMI/15% HS/glutamine/antibiotics. All cells were maintained at 37°C in a humidified incubator containing 6% CO_2_ and were passaged every 3–4 days. For the experiments described here, cells were grown from a single frozen vial of each cell type.

### Preparation of Hp containing LTCs

Haematoporphyrin hydrochloride (0.11 mmol) was dissolved in 10 ml chloroform and activated by addition of 0.173 mmol of *N*-hydroxysuccinimide and 0.11 mmol DCC. The mixture was stirred at room temperature for 2.5 h. Following evaporation with a stream of air, the residue was dissolved in 2 ml of tetrahydrofuran (THF) and the activated Hp was slowly added to a solution of 15 mg transferrin dissolved in 10 ml of 0.1 M NaHCO_3_ cooled on ice. The solution was allowed to warm to room temperature, adjusted to pH 7.5 and stirred vigorously overnight. Tubes containing Hp were protected from light exposure. The conjugate solution was centrifuged (7200 **g**, 30 min, 4°C) and the supernatant was analysed spectroscopically for the content of protein (*λ*=280 nm) and Hp (*λ*=400 nm). After dialysis, a small portion of the crude reaction product was chromatographed over Sephadex G-50 equilibrated with 5 mM NaHCO_3_ (pH 8.0) or 10 mM phosphate-buffered saline (PBS) (pH 7.2). Fractions containing materials with absorption peaks at 280 and 400 nm were collected and stored at 4°C.

### Characterisation of the Tf–Hp conjugate

#### High-pressure liquid chromatography

Tf, Hp and Tf–Hp were chromatographed over a C-18 column (3.9 × 300 mm Bondclone, particle diameter 10 *μ*m, Phenomenex, Torrance, CA, USA) using a HPLC JASCO-1580 with a JASCO 1575 UV/VIS detector set at 280 nm. The solvent system was composed of acetonitrile–water with 1% trifluoroacetic acid and compounds were eluted with a linear gradient (20–100% acetonitrile).

#### Absorbance spectrum

The absorbance spectra of PBS solutions of Hp (0.02 mg ml^−1^), Tf (1.4 mg ml^−1^) and Hp-Tf (1.4 mg ml^−1^) were recorded with a CHEMUSB2-UV-VIS spectrophotometer having optical resolution of 1 nm, grating of 600 lines mm^−1^ equipped with CCD array detector. The samples were scanned in the absorbance region of 250–500 nm.

#### Biological activity

This was assessed by the ability of antitransferrin or anti-BSA antibodies to inhibit PDT-induced cytotoxicity. Friend's leukaemia cells were cultured with 3 *μ*M Tf–Hp in the LTC cytotoxicity assay (see below) together with 0–200 *μ*g ml^−1^ of each antibody.

### Ligand toxin conjugate cytotoxicity assay

Late log-phase cells were washed with DMEM prewarmed to 37°C and cultured at 0.5–1 × 10^5^ cells ml^−1^ either alone or with increasing concentrations of Tf–Hp LTC for 2 h at 37°C in 6% CO_2_. Cells were then washed with DMEM, exposed to white fluorescent light (fluence rate=0.5 mW cm^−2^) overnight, usually 16 h and then recultured in full medium for 24 h. The emission spectrum covered the wavelengths 350–700 nm. Cell viability was determined by trypan blue exclusion. Experiments were repeated at least three times. The optimal exposure times of cells to the Ps and fluorescent radiation were determined in preliminary time-course experiments (data not shown).

### Fluorescence microscopy of Hp and Tf–Hp endocytosis

Friend's leukaemia cells were grown on glass slides in tissue culture dishes together with Hp or Tf–Hp and the incorporated fluorescence was followed at various time intervals with an AX70 Olympus microscope equipped with a high-pressure mercury lamp for excitation and a set of filters for blue violet excitation (band path 420–480 nm), dichroic mirror (455 nm) and a cut-on red emission barrier filter (580 nm). Fluorescence was analysed with an 60 × objective, without addition of any antibleaching solution, and recorded by a CCD camera.

### Cellular uptake of Tf–Hp conjugate

To ascertain the kinetics of cellular uptake of the Tf–Hp conjugate, 10^6^ ml^−1^ FL cells were incubated at 37°C for 0, 15, 30, 60 or 120 min with 3 *μ*g ml^−1^ Tf–Hp. The cells were then washed three times with PBS and the fluorescence was measured by FACSCalibur (Beckon Dickinson, San Jose, CA, USA). Approximately 10^4^ cells were analysed in each sample. From the output data, the increase in mean fluorescence units per minute was calculated.

### Resistance to Tf–Hp treatment

To test the effect of repeated exposure to Tf–Hp, FL cells were treated with Tf–Hp in the cytotoxicity assay as described above. Cells surviving treatment at the LD_90_ concentration were maintained in Tf–Hp-free culture medium until they regained pretreatment growth kinetics (approximately 3 weeks). These cells were designated ‘*Single treatment FL.*’ and together with previously untreated FL, were tested for their response to a range of LTC doses. Single-treatment FL cells surviving a second exposure at LD_90_ were in turn cultured and termed ‘*Double treatment FL.*’ This cycle was repeated to derive ‘*Triple treatment FL.*’

### Measurement of intracellular CL induced by luminol

To ascertain the ability of luminol to induce intracellular luminescence, we performed measurements using a fluorescence spectrometer (Varian Eclipse, Palo Alto, CA, USA) set at bio/CL mode. Stock solutions of 100 mM luminol in 10% NaOH and 5 mM ferrous sulphate and LB buffer (0.14 M NaCl, 0.027 M KCl, 0.012 M Na_2_HPO_4_.2H_2_O, 0.015 M K_2_PO_4_, 0.9 mM CaCl_2_, 0.5 mM MgCl_2_.6H_2_0, pH=7.6, Kawagoe and Nakagawa, 2000) were prepared. A mixture of 1 mM luminol, 1 mM iron catalyst and 1% H_2_O_2_ was also prepared and used to calibrate the system. Then to measure the influence of luminol on cellular luminescence, log-phase FL cells were collected, washed twice in LB buffer, resuspended at 10^5^ or 10^6^ viable cells ml^−1^ in LB buffer+1 mM iron catalyst alone or together with 1 mM luminol. The cell suspensions were monitored for CL for up 50 min.

### Intracellular PDT activation by luminol

In initial experiments, FL cells were washed and cultured for 20 h with different concentrations of Hp or Tf–Hp (0.07, 0.15 or 0.3 *μ*M) together with luminol (0–10 *μ*M). All manipulations of cells and components were performed with the room lights switched off. Culture plates were wrapped in aluminium foil during the culture period. Subsequent experiments aimed to test whether a PDT effect could be obtained by staggering the exposure of the cells to either luminol or Tf–Hp conjugate. In one series, FL cells were cultured for 2 h in the dark at 37°C with Tf–Hp (3 *μ*M), washed and resuspended in medium at the standard culture concentration. This procedure took approximately 15 min. Then, the cells were kept at 37°C for an additional 0, 30, 60 or 90 min, luminol (10 *μ*M) was added and the cultures incubated in the dark for a further 16 h. Alternatively, cells were first cultured for 24 h in the dark in the presence of 10 *μ*M luminol, washed or not-washed and then cultured further for 24 h in the presence of Tf–Hp (0–3 *μ*M) at 37°C. During washing procedures and cell handling, special care was taken to maintain the cells in a dark environment.

### Statistical analysis

Statistical analyses of the data were performed by two-way analysis of variance (ANOVA) (F-distribution) and least-squares regression.

## RESULTS

### Purification and characterisation of the Tf–Hp conjugate

**Figure 1:**
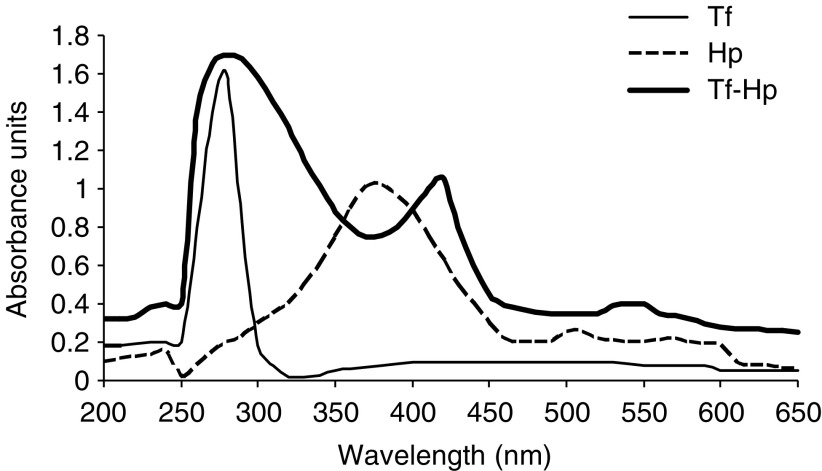
UV-VIS absorbance spectra in the interval of 250–500 nm for Hp (- - - - - -), Tf (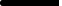) and Hp-Tf (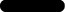) prepared in PBS (for concentrations see Materials and Methods).

The Tf–Hp conjugate was separated from Tf and Hp by HPLC and characterised by UV-Vis spectrophotometry (Figure 1). The Tf spectrum reveals a typical maximum at *λ*=280 nm, whereas Hp absorption maximum is at *λ*=375 nm. The Hp–Tf conjugate revealed two absorption peaks at *λ*=280 and 412 nm and the spectrum is characterised by a red shift of the maximum and is not a simple superposition of the spectra of its components. The molar ratio of the two conjugates components was determined spectroscopically to be 6.3 Hp/1 Tf. The biological activity of the conjugate was verified by determining the ability of antibodies to interfere with LTC cytotoxicity. The addition of antitransferrin to assay cultures of FL cells reduced cytotoxicity by 50% at 100 *μ*g ml^−1^ antibody and by 95% at 200 *μ*g ml^−1^ antibody. The addition of anti-BSA to parallel cultures reduced cytotoxicity by 10 and 5%, respectively.

### Direct cytotoxicity of Tf–Hp LTCs with external photoactivation

**Figure 2:**
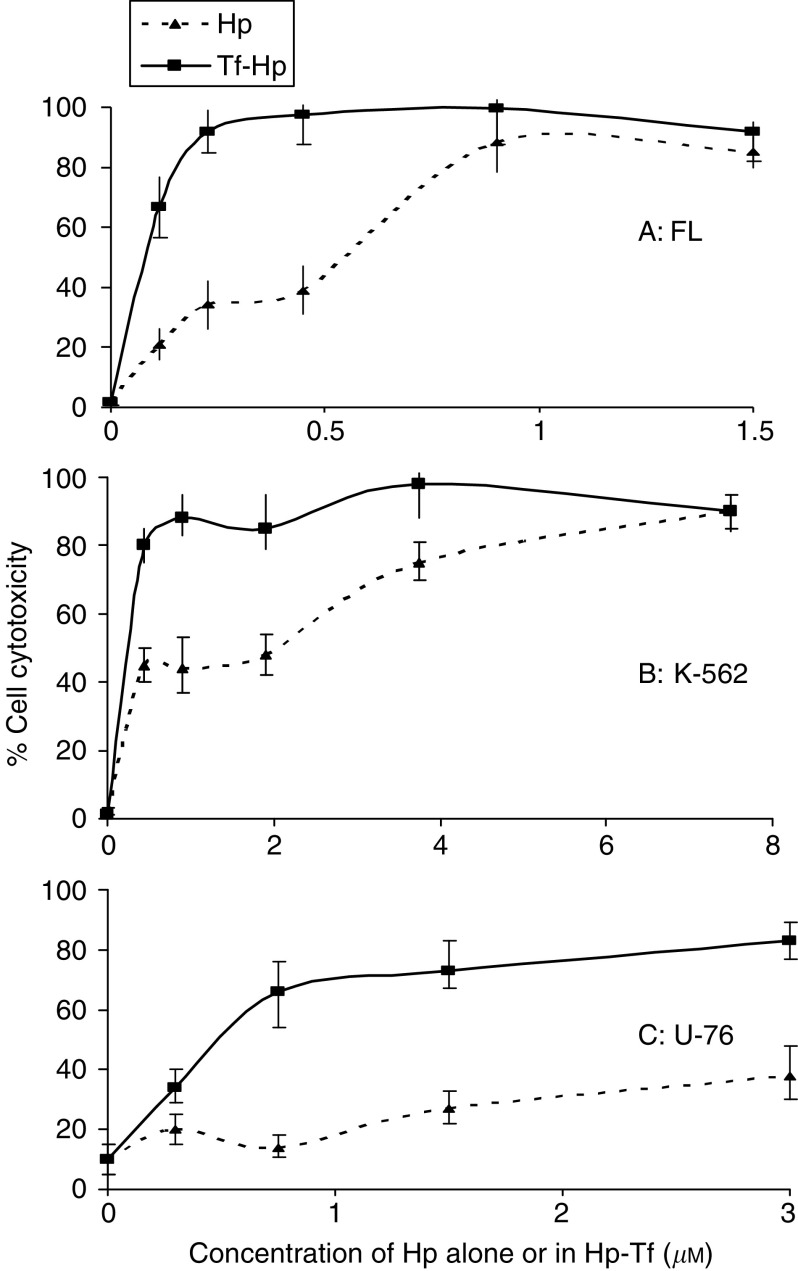
Dose-dependent cytotoxicity of Hp and Tf–Hp for cells FL (**A**), K562 (**B**) and U-7.6 (**C**) cells. 0.5–1 × 10^5^ cells ml^−1^ were cultured for 2 h in the dark in medium alone or containing Hp or Tf–Hp (0–3 *μ*M), washed, exposed to ambient fluorescent light for 16 h at room temperature and then recultured in full medium for 24 h. Cell viability was assessed by trypan blue exclusion. The data represent the mean and standard deviation from at least three experiments.

Friend's leukaemia, K562 and U-76 cells were incubated with various concentrations of Hp or Tf–Hp in the dark for 2 h and then exposed overnight exposure to white fluorescent light. Control cells kept under similar environmental conditions but not exposed to Hp or light showed 95% viability. Treatment with high concentrations of Tf–Hp alone induced low-level cytotoxicity (Figure 7). For all cell types, dose–response effect (Figure 2) of Tf–Hp was significantly more cytotoxic than Hp alone (for FL *P*=0.042; for K-562 *P*=0.034, for U-76 *P*=0.038). Table 1 shows that the concentration of Tf–Hp required to achieve LD_50_ was more than six-fold lower than for Hp. Furthermore LD_100_ values were only obtained with the Tf–Hp. U-76 hybridoma cells were relatively insensitive to PDT. The concentration of Tf–Hp required to reach LD_90_ in these cells was >19.4-fold higher than for FL cells and >3.5-fold higher than for K-562 cells. This order of sensitivity was retained at the concentrations required for LD_MAX_ (3.37 for FL and 0.8 for K562). Furthermore, 100% cytotoxicity was only obtained when the conjugate was used against the erythroleukemic cell lines. A similar pattern of sensitivity was also seen with free Hp treatment. Although a similar (90%) LD_MAX_ was reached for both erythroleukemic lines, FL cells were 16.6 more sensitive than K-562.

**Figure 3:**
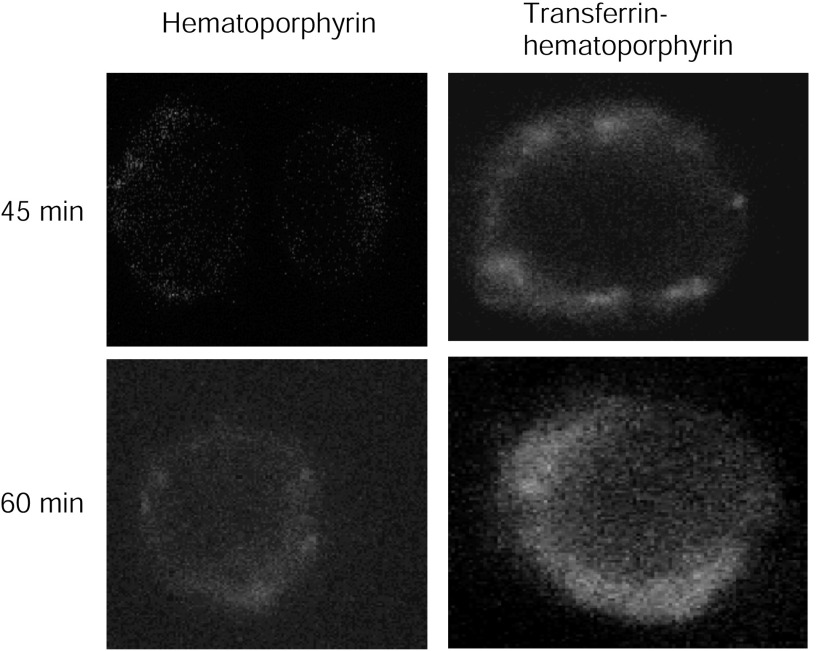
Fluorescent microscopy of Hp and Tf–Hp-treated FL cells. Cells were grown on glass slides in tissue culture dishes together with medium containing 1 *μ*M of Hp or Tf–Hp for 45 and 60 min and then examined for fluorescence emission. Hp was found in relatively low levels around the cytoplasmic membrane whereas Tf–Hp was mainly internalised in intracellular vesicles.

Further evidence for the increased cytotoxicity of Tf–Hp over free Hp was obtained from fluorescence microscopy. Figure 3 illustrates the presence and location of the Ps in FL cells after 45 and 60 min incubation with either Hp (left panel) or Tf–Hp (right panel). At both time points, relatively faint (as no anti bleaching solution was used) Hp fluorescence was observed mainly constrained to the plasma membrane region. Significantly greater fluorescence was apparent in cells treated with Tf–Hp. After 45 min, the conjugate localised in membrane patches (probably demarcating endolysosomal compartments) and had infiltrated much of the cytoplasm by 60 min.

**Figure 4:**
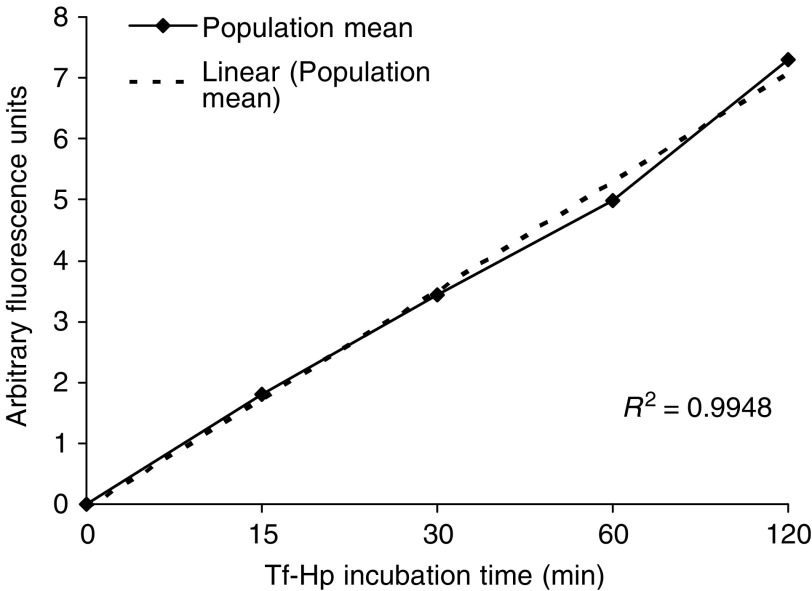
Kinetics of Tf–Hp uptake into FL cells. 106 cells were incubated with 3 *μ*g Tf–Hp for the times indicated. Cells were washed three times with PBS and fluorescence measured by FACS. The figure depicts the increase in the mean arbitrary fluorescence units measured for the total cell sample measured (approx. 103 cells at each time period) *vs* incubation time. The least-squares linear regression line also shown.

The characteristics of Tf–Hp uptake kinetics in the FL were examined by incubating them with conjugate for various times and measuring the cell-associated fluorescence by fluorescence-activated cell sorting (FACS). Figure 4 shows that the uptake was linear over the 120 min of the experiments with an uptake rate of 0.056 arbitrary fluorescence units min^−1^.

### Resistance of FL cells to repeated Tf–Hp treatments

**Figure 5:**
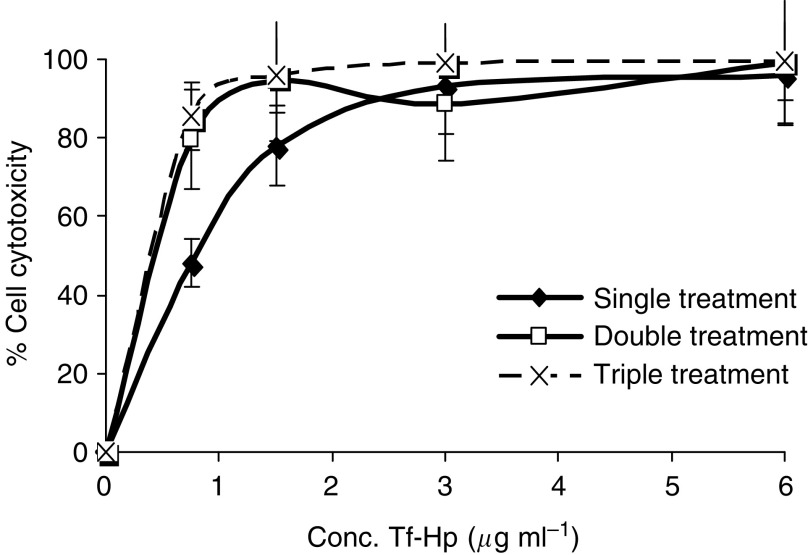
Repeated exposure to Tf–Hp does not induce resistance in treated cells. Friend's leukaemia cells were treated with Tf–Hp for dose–response (see Materials and Methods) (◊). Cells surviving treatment at the LD_90_ concentration were cultured in Tf–Hp-free culture medium for approximately 3 weeks and then re-tested, together with fresh FL cells, for their dose–response to Tf–Hp challenge (▪). Cells surviving this second treatment at the LD_90_ were processed through a third treatment cycle (** × **). Cell viability was determined by trypan blue exclusion. Data points represent the mean±standard deviation of three experiments. Photodynamic therapy sensitivity of FL cells was significantly increased after repeated exposure to Tf–Hp at 0.75 *μ*g ml^−1^ Tf–Hp (*P*<0.009) and at 1.5 *μ*g ml^−1^ Tf–Hp (*P*<0.012).

We tested whether repeated exposure to Tf–Hp may lead to the development of resistance in treated cells by adapting a protocol used previously by us to study resistance to a ligand–Ricin A conjugate (Firer *et al*, 2003). Figure 5 shows the dose–response curve of FL cells exposed to a range of Tf–Hp concentrations (first treatment). Those surviving the LD_90_ dose were passaged over several weeks in Tf–Hp-free medium and then retested for their dose–response to Tf–Hp (second treatment). This process was repeated (third treatment). This protocol of repeated exposure did not result in the development of resistant cells as would be demonstrated by a shift to the right in the dose–response curve. Indeed, statistical analysis by ANOVA at different Tf–Hp concentrations showed that repeated treatment of FL cells resulted in a statistically significant elevation in PDT sensitivity at 0.75 *μ*g ml^−1^ Tf–Hp (*P*<0.009) and at 1.5 *μ*g ml^−1^ Tf–Hp (*P*<0.012). At 3 *μ*g ml^−1^ Tf–Hp, there was no significant difference between the first, second or third treatments.

### Intracellular CL by luminol

**Figure 6:**
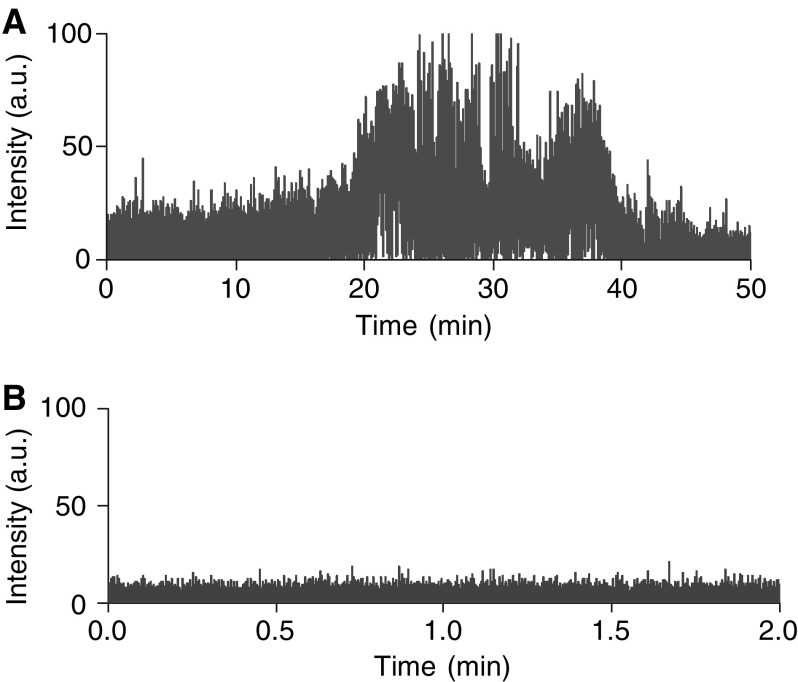
Intracellular CL in FL cells induced by luminol. Cells were suspended in LB buffer (see Materials and Methods)+1 mM iron catalyst and used to measure background CL (graph **A**) over a 50 min incubation period. A separate aliquot of cells were similarly suspended but together with 1 mM luminol (graph **B**). Measurements were made with a fluorescence spectrometer set to bio/CL mode and the readout was in arbitrary units.

Experiments were performed to determine whether luminol is capable of inducing intracellular CL. Friend's leukaemia cells were incubated with a source of catalyst (ferrous sulphate) with or without luminol and CL measurements were made using a fluorescence spectrometer. Figure 6 shows that incubation of cells with catalyst did produce notable CL over a 50 min incubation period. However, addition of luminol to the cell suspension resulted in measurable luminescence after about 20 min incubation that peaked after an additional 10 min.

### Intracellular PDT activation of Hp or Tf–Hp by luminol

**Figure 7:**
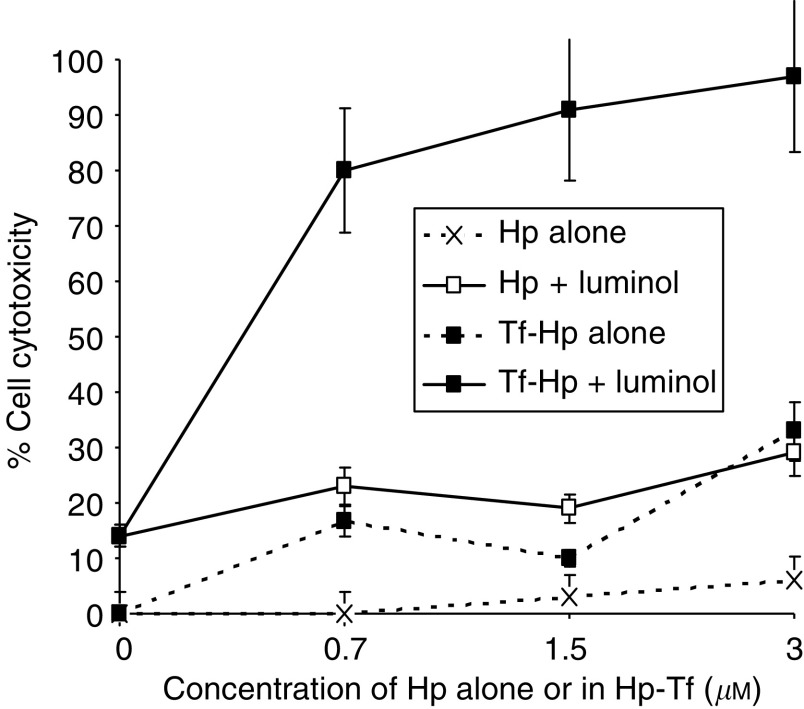
Molecular, intracellular activation of the PDT effect. FL cells were cultured in the dark for 48 h at 37°C with haematoporphyrin (Hp) or Tf–Hp conjugate (0–3 *μ*M) together with 10 *μ*M luminol. No other manipulation or external radiation of the cells was performed. At the end of the culture period, cell viability was determined by trypan blue exclusion.

**Figure 8:**
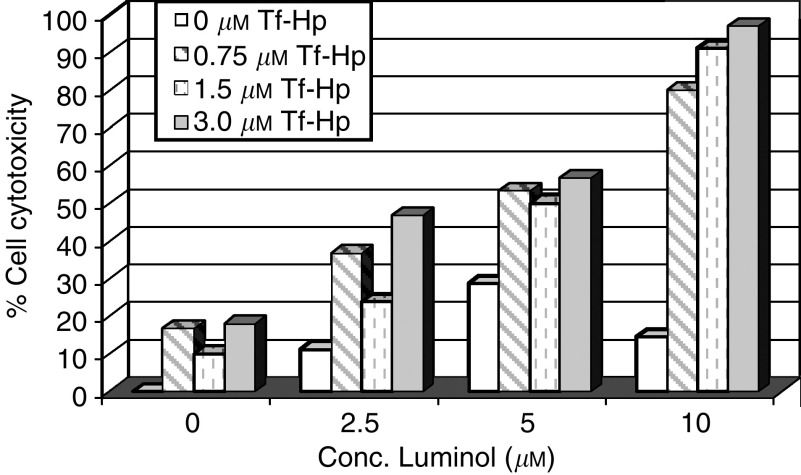
The effect of component concentration of the IAP. Friend's leukaemia cells were cultured in the dark for 48 h at 37°C with varying concentration combinations of Tf–Hp conjugate (0–3 *μ*M) and luminol (0–10 *μ*M). No other manipulation or external radiation of the cells was performed. At the end of the culture period, cell viability was determined by trypan blue exclusion.

**Figure 9:**
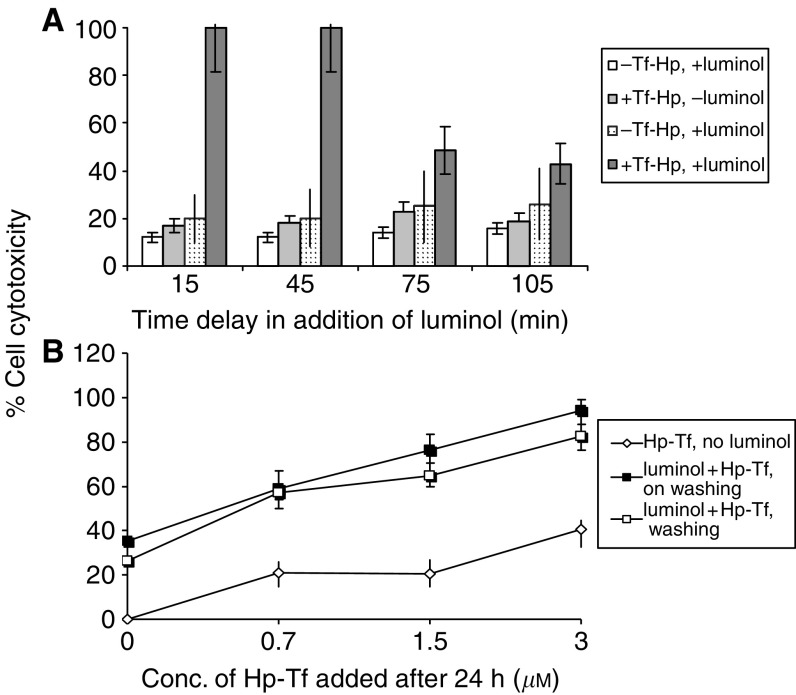
The molecular components for IAP need not be added in synchrony. (**A**) Effect of delayed PDT activation by luminol on cytotoxicity of Tf–Hp treated FL cells. The cells were cultured for 2 h in the dark at 37°C with Tf–Hp (3 *μ*M) and washed. After various delay times (0, 30, 60 or 90 min), luminol (5 *μ*M) was added and the cells were cultured for a further 16 h in the dark. The figure shows the % of induced cytotoxicity. The 0 delay point includes time for washing of cells and returning them to the culture (approximately 15 min). (**B**) Effect of pretreatment of FL cells by luminol on cell growth inhibition by Tf–Hp. The cells were first cultured for 24 h in the presence of 10 *μ*M luminol in the dark for 24 h, washed or nonwashed and then cultured for a further 24 h in the presence of Tf–Hp (0–3 *μ*M) at 37°C.

Despite the effective action of the Tf–Hp, PDT for leukemia would be limited *in vivo* by the restricted tissue penetration of candescent light. We therefore tested the possibility of generating an intracellular chemiluminescent light signal to induce PDT. Figure 7 illustrates the cytotoxicity induced in FL cells cultured in the dark with Hp or Tf–Hp either alone or together with 10 *μ*M luminol as the chemiluminescent energy source. The cells were not exposed to ambient fluorescent light at any stage of the procedure. We found that (i) luminol alone induced about 15% cytotoxicity, (ii) Hp alone had little effect on cell viability, (iii) cytotoxicity reached a maximum of 30% in the presence of Hp and luminol and (iv) luminol induced a significant PDT effect upon addition of Tf–Hp (*P*=0.0043). Figure 8 further demonstrates that the cytotoxic luminol-induced PDT effect is dependent on the concentration of both Tf–Hp and luminol with a combination of 10 *μ*M luminol and 3 *μ*M of conjugate producing maximum cytotoxicity. A reduction in Tf–Hp concentration had less effect on cytotoxicity than did diluting the level of luminol. We tested if synchrony in exposure to luminol and Tf–Hp is a requirement for this cytotoxicity by incubating cells first with Tf–Hp, washing and then exposing them to luminol following various delay times. The time taken to wash the cells and return them to culture was approximately 15 min. Although delaying the exposure to luminol by 30 min had no effect on the cytotoxicity (Figure 9A), after 60 min of delay, the PDT effect was reduced by 50%. However by reversing the protocol (Figure 9B), we found that preincubation with luminol for 24 h sensitised the cells to the delayed exposure to Tf–Hp and the PDT effect was dose dependent. Washing or not washing the luminol-incubated cells before their exposure to Tf–Hp had no significant effect on the IAP activity.

## DISCUSSION

This study addresses two aspects of PDT technology. The first concerns the development of PDT systems to enhance the efficiency of delivery to target cells. Most (Hamblin and Newman, 1994; Klyashchitsky *et al*, 1994; Dolmans *et al*, 2003), but not all (Gijsens *et al*, 2002; Sharman *et al*, 2004) targeted PDT studies have used monoclonal antibodies as the address moiety. As the use of antibodies poses several practical limitations (Rihova, 1998), an alternate approach is to target a Tf–Ps conjugate to Tf receptors that are overexpressed on tumour cells (Faulk *et al*, 1980; Cavanaugh, 2002). The therapeutic potential of Tf–protein (Weaver and Laske, 2003) and Tf–chemical (Singh *et al*, 1998) toxin conjugates have already been examined, but less is known about Tf–Ps conjugates particularly with regard to Hp which, although having been used successfully in free form in the clinic for over a decade (Dolmans *et al*, 2003; Brown *et al*, 2004), has been little tested in targeted PDT (Hamblin and Newman, 1994; Gijsens *et al*, 2002).

We prepared and characterised Tf–Hp conjugates and found that these were at least six-fold more effective in inducing cell death at the LD_50_ level (Figure 2 and Table 1)_._ High concentrations of Tf–Hp alone induced low-level cytotoxicity in accordance with previous reports (Supino *et al*, 1986; Luksiene and de Witte, 2003), an effect that may be related to the ability of Hp to inhibit the activity of protein kinase C (Luksiene and de Witte, 2003). However, there was variability among the cells in their sensitivity to PDT, with erythroleukemic cells showing between 3.5 and 19.4-fold greater sensitivity than hybridoma cells. We have seen a similar relative lack of PDT sensitivity in other hybridoma cells (data not shown) and, although we did not measure this parameter, we believe that it reflects the high TfR levels on erythroid cells. A similar pattern of sensitivity was also seen with free Hp treatment. Because in order to induce cytotoxicity Hp must interact with cell membrane lipids (Ehrenberg *et al*, 1985), the results suggest that the molecular makeup of the three cell membranes is different. This assumption is supported by earlier studies showing that variation in lipid bilayer components, in particular cholesterol and phosphatidylcholine, influences the binding and depth of penetration of Hp into cell membranes and liposomes (Lavi *et al*, 2002). Consideration should also be made as to the relative contributions of Tf and Hp to the overall contact between the conjugate and the cell membrane. We do not know the spatial orientation of the two components within the conjugate but the observation that in FL cells for example, a three-fold increase in cytotoxicity was obtained with Tf–Hp as compared to free Hp, suggests that the Tf makes a foremost contribution to the conjugate–membrane interaction and/or the effectiveness of its uptake.

Aside from increasing target specificity and efficiency, PDT-induced cell death is faster when Tf–Hp is used. For example, during optimisation of the LTC cytotoxicity assay we noticed that whereas almost 100% cytotoxicity was achieved after only 30 min exposure to Tf–Hp, about 2 h were required for maximum activity (24%) of free Hp (data not shown). Moreover, fluorescence microscopy of Hp and Tf–Hp-treated FL cells (Figure 3) demonstrated that Tf–Hp is taken up more rapidly and that it reaches intracellular organelles and this would provide for more effective disruption of intracellular membranes. This is relevant in light of data demonstrating that ^1^O_2_ molecules generated by Ps activation rapidly diffuse out of the cell surface bilayer (Lavi *et al*, 2002).

An important limitation of current chemotherapy is the frequent induction of multidrug resistance (MDR) in treated cells to a range of compounds that share apparently no structural or functional similarities (Hirose, 2002; Liscovitch and Lavie, 2002). Several cell surface transporter proteins have been identified as central components in this phenomenon (Gottesman *et al*, 2002). Interestingly, we (Firer *et al*, 2003) and others (Wang *et al*, 2000; Mazel *et al*, 2001) have shown that LTCs comprising protein toxins or DNA-damaging drugs seem to bypass the MDR transporters and do not to induce resistance. This is probably due to the pathway of cell entry that involves receptor-mediated endocytosis, initial passage of the LTC to lysosomes and then release of the toxin component to other intracellular targets, such as the endoplasmic reticulum and nucleus. With regards to PDT, whereas this modality has been found effective against MDR tumour cells (Capella and Capella, 2003; Preise *et al*, 2003), very few studies have directly approached the possibility of resistance developing to repeated Ps exposure. Madsen *et al* (2003) for instance reported that no resistance developed in glioma spheroids repeatedly exposed to free 5-aminoelulinic acid but as we have shown that LTCs are much more effective than free Ps we subjected FL cells to several rounds of treatment with suboptimal doses of Tf–Hp (Figure 5). Our results support Madsen *et al*'s conclusion that PDT does not induce the MDR phenomenon. This is in contrast to the well recognised resistance that FL cells develop to repeated exposure to other cytotoxins such as anthracyclines (Abbadessa *et al*, 1992; Desoize *et al*, 1994). From the fluorescence microscopy results (Figure 3), it may be postulated that LTC treatment concentrates the Ps in intracellular organelles that are not available to MDR transporters. The data from Figure 5 further indicate that FL cells surviving an initial LTC-PDT attack become more sensitive to subsequent treatments. At this point, we do not have an explanation for this phenomenon.

The second issue of PDT technology we addressed concerned the source of the luminescent activating signal delivered to the Ps. External radiation provides homogeneous excitation of Ps to cells in tissue culture or those injected subcutaneously; however, penetration of visible spectrum light waves into internal tissue is not uniform for all wavelengths (Moan and Peng, 2003) as after only a few millimeters, the cellular mass causes a dramatic drop in and scattering of transferred energy, precluding the use of PDT for deeper tissue targets (Ceburkov and Gollnick, 2000). Efforts to overcome this limitation have concentrated on new external light devices (Brancaleon and Moseley, 2002; Chen *et al*, 2002) or improved catheters. Our aim was to provide a molecular light-emitting mechanism within or at least proximal to the Ps-loaded target cell. This strategy is noninvasive, does not expose normal tissue to irradiation and a molecular illuminator could potentially be transported to any target cell *in vivo*. We use the term *intracellular activation of PDT* (*IAP*) to describe such molecular systems.

One CL activator is luminol, which undergoes a light-emitting process catalysed by metal ions and hydrogen peroxide (White *et al*, 1964; Weeks, 1992). This process is extensively employed in chemiluminescent detection techniques (Kricka, 2002; Templin *et al*, 2002) and in cell physiology studies (Kubo *et al*, 1987; Nemeth *et al*, 1999; Kawagoe and Nakagawa, 2000) but there is no literature on the use of luminol as an energy source in the field of PDT of cancer cells. The emission spectrum of luminol has been reported to comprise two major peaks, at 424 and 485 nm (White *et al*, 1964). We found that the first of these corresponds to a crest in the absorption spectrum of Tf–Hp (412 nm) but not to those of free Hp or Tf (375 and 280 nm, respectively, Figure 1). This information, together with the enhanced intracellular uptake of the Tf–Hp relative to Hp suggested to us that an IAP involving luminol might be effective for our system.

Firstly, we determined that luminol could indeed induce intracellular CL. Figure 6 demonstrates that after a period of incubation, cells exposed in the dark to luminol and an iron catalyst luminescence. This response was not of rapid onset that might reflect slow cellular uptake of luminol. In subsequent experiments, Hp or Tf–Hp was mixed with luminol and added to FL cell culture in the dark (Figure 7). Not only was there a significant PDT effect when both Tf–Hp and luminol were added to the cells, but the cytotoxic efficiency of Tf–Hp over Hp was even more enhanced than that seen with the external light source (Figure 2). However, the concentration of Tf–Hp required to attain LD_MAX_ in the IAP system was 6.7 times higher than with external radiation (Figure 7 and Table 1). Luminol alone appeared to induce little cytotoxicity and these levels were similar to the PBS control (data not included in the figure). As this IAP system progresses to *in vivo* studies one important parameter to test will be the physiological effect, in any, of luminol. Little information is currently available on this issue, although a recent study by Sanders *et al* (2000) demonstrated that the compound had no detrimental effect on rat metabolism.

Luminol-induced CL has been used in the past to study physiological parameters of phagocytic cells, as these produce the H_2_O_2_ needed for luminol oxidation (Nemeth *et al*, 1999; Dunn *et al*, 2005). But nonphagocytic cells, such as those used in this study, also possess several potential pathways through which to generate luminol-activating H_2_O_2_. These include a variety of signalling pathways (Finkel, 1998) and intracellular iron metabolism (Caltagirone *et al*, 2001). Also, reactive oxygen species such as O_2_^*^ produced in mitochondria by the electron transport chain are dismuted to H_2_O_2_ (Batandier *et al*, 2002), which diffuses into the cytoplasm (Antunes and Cadenas, 2000). Oxidant products of nitric oxide have also been shown to activate luminal (Catz *et al*, 1995).

Other IAP systems have also been studied. Carpenter *et al* (1994) described a bioluminescent mechanism for PDT that induced killing of virus-infected cells, involving the activation of hypericin following oxidation of luciferin by luciferase. They too reported that a continuous source of external radiation resulted in more effective target cell cytotoxicity and suggested that this may be related to suboptimal proximity between the luciferin and the hypericin. An alternate explanation based on the short half-life of the CL reaction itself (∼1 min) and rapid decrease in light emission intensity cannot be ruled out. Whatever the explanation, these limitations might be overcome by incorporating enhancer molecules into the IAP system to prolong and intensify the luminescent signal analogous to improvements made to the original chemiluminescent techniques (Kricka, 2000).

Additional experiments (Figure 9) demonstrated that the Ps and IAP systems need not be applied simultaneously in order to produce an effective PDT response. Delaying the addition of luminol (by up to 45 min) to Tf–Hp-loaded cells, or delaying the addition of Tf–Hp (24 h) to luminol-loaded cells did not affect cytotoxicity. As the loaded cells were thoroughly washed before the exposure to luminol, these results reflect activation of the internalised Tf–Hp or luminol rather than material loosely bound to the membrane.

In conclusion, our data demonstrate that Tf–Hp conjugates represent an effective vehicle for PDT-induced cytotoxicity. Transferrin conjugates are being clinically tested against neurological tumours (Hall, 2000) and it remains to be seen whether the presence of Tf receptors on normal cells will preclude the use of Tf–Hp against haematological cancers (Hamblin and Newman, 1994; Brown *et al*, 2004). Experience with monoclonal antibodies against nontumour-specific antigens such as CD20 and EGFR (Tobinai, 2002; Goldberg, 2005) suggests that the enhanced expression of TfR on tumour cells and the targeting effect of Tf–Hp may provide an effective therapeutic window. We are currently studying this with *in vivo* murine models. Finally, the ability to activate the PDT effect intracellularly without the need for external radiation opens the way for wider application of PDT technology, particularly where internal diseased tissues are the target.

## Figures and Tables

**Table 1:** Comparison of cytotoxic efficiency of PDT induced by Tf–Hp or Hp for three cell lines

	**Cell type**		
**Parameter**	**FL**	**K-562**	**U-7.6**
*Concentration at LD*_*50*_ (*μM*)			
Hp	0.55	1.9	NR
Tf–Hp	0.08	0.3	0.5
Ratio^a^	6.88	6.33	—
			
*Concentration at LD*_*90*_ (*μM*)			
Hp	NR	7.5	NR
Tf–Hp	0.18	1.0	>3.5
Ratio^a^	—	7.5	—
			
*Concentration at LD*_*MAX*_ (*μM*)			
Hp	0.89	7.5	3.0
Tf–Hp	0.45	3.75	3.0
Ratio^a^	1.98	2.0	1.0
			
% *Cell cytotoxicity at LD*_*MAX*_			
Hp	84	90	40
Tf–Hp	100	100	85
Ratio^a^	0.84	0.90	0.47

a Ratio of Hp/Tf-Hp.

NR=not reached.
